# Induction of Porcine Host Defense Peptide Gene Expression by Short-Chain Fatty Acids and Their Analogs

**DOI:** 10.1371/journal.pone.0072922

**Published:** 2013-08-30

**Authors:** Xiangfang Zeng, Lakshmi T. Sunkara, Weiyu Jiang, Megan Bible, Scott Carter, Xi Ma, Shiyan Qiao, Guolong Zhang

**Affiliations:** 1 Department of Animal Science, Oklahoma State University, Stillwater, Oklahoma, United States of America; 2 State Key Laboratory of Animal Nutrition, China Agricultural University, Beijing, China; 3 Department of Biochemistry and Molecular Biology, Oklahoma State University, Stillwater, Oklahoma, United States of America; 4 Department of Physiological Sciences, Oklahoma State University, Stillwater, Oklahoma, United States of America; Iowa State University, United States of America

## Abstract

Dietary modulation of the synthesis of endogenous host defense peptides (HDPs) represents a novel antimicrobial approach for disease control and prevention, particularly against antibiotic-resistant infections. However, HDP regulation by dietary compounds such as butyrate is species-dependent. To examine whether butyrate could induce HDP expression in pigs, we evaluated the expressions of a panel of porcine HDPs in IPEC-J2 intestinal epithelial cells, 3D4/31 macrophages, and primary monocytes in response to sodium butyrate treatment by real-time PCR. We revealed that butyrate is a potent inducer of multiple, but not all, HDP genes. Porcine β-defensin 2 (pBD2), pBD3, epididymis protein 2 splicing variant C (pEP2C), and protegrins were induced markedly in response to butyrate, whereas pBD1 expression remained largely unaltered in any cell type. Additionally, a comparison of the HDP-inducing efficacy among saturated free fatty acids of different aliphatic chain lengths revealed that fatty acids containing 3–8 carbons showed an obvious induction of HDP expression in IPEC-J2 cells, with butyrate being the most potent and long-chain fatty acids having only a marginal effect. We further investigated a panel of butyrate analogs for their efficacy in HDP induction, and found glyceryl tributyrate, benzyl butyrate, and 4-phenylbutyrate to be comparable with butyrate. Identification of butyrate and several analogs with a strong capacity to induce HDP gene expression in pigs provides attractive candidates for further evaluation of their potential as novel alternatives to antibiotics in augmenting innate immunity and disease resistance of pigs.

## Introduction

Infectious diseases continue to pose significant economic losses to the animal industry. The routine use of antibiotics at subtherapeutic doses for disease prevention in animal production practices is suspected to be a major force that drives the emergence of antibiotic-resistant pathogens [1], [2]. Therefore, it is imperative to develop novel antimicrobial strategies with a low risk of triggering resistance. Dietary compounds with the capacity to augment HDP synthesis and host immunity are attractive candidates as alternatives to antibiotics for disease control and prevention [3]. As a critically important first line of defense, HDPs are produced mainly by both mucosal epithelial cells and phagocytes in vertebrate animals [4], [5]. These peptides are broadly active against a range of pathogens including antibiotic-resistant strains. Unlike conventional antibiotics with no immune regulatory functions, HDPs are capable of modulating the host immune system by recruiting and activating different types of immune cells [6]. Additionally, most HDPs have the capacity to dampen the bacterial infection-induced inflammatory response, because of their strong affinity for bacterial membrane components such as lipopolysaccharides [7].

Defensins and cathelicidins represent two major families of HDPs in vertebrate animals [8], [9]. Multiple members of each family generally exist in most animal species. For example, 13 β-defensins and 11 cathelicidins have been reported in pigs [10], [11]. Porcine β-defensin 1 (pBD1), pBD2, pBD3, pBD129, and epididymis protein 2 splicing variant C (pEP2C) are expressed in a wide range of tissues, whereas pBD4, pBD123, and pBD125 are primarily restricted to the male reproductive tissues [12], [13]. Eleven members of porcine cathelicidins include proline/arginine-rich peptide of 39 amino acids (PR-39), cysteine-rich protegrins (PG) 1–5, proline/phenylalanine-rich prophenins (PF) 1–2, and porcine myeloid antimicrobial peptides of 23, 36, and 37 amino acids, also known as PMAP-23, PMAP-36, and PMAP-37, respectively [10], [11]. Porcine cathelicidins are mainly derived from bone marrow myeloid cells, albeit with a restricted expression in many other tissues [10], [11].

Besides infection and inflammation, HDPs have been found to be specifically regulated by several different dietary compounds. For example, vitamin D_3_ is a potent inducer of human cathelicidin LL-37 [14], [15]. Several di- and monosaccharides such as lactose, trehalose, maltose, glucose, and galactose were revealed recently to stimulate LL-37 synthesis in human cells [16]. Essential amino acids including isoleucine and arginine are capable of enhancing HDP expression in humans, cattle, and pigs as well [17]–[20]. A trace mineral, namely zinc, has been found to augment HDP expressions in both human and porcine intestinal epithelial cells [20], [21]. Moreover, probiotic lactobacilli potentiate HDP expression in human cells and piglets [22], [23]. Butyrate, a major species of short-chain fatty acids (SCFAs) that are produced by bacterial fermentation of undigested dietary fiber in the large intestine, has a strong capacity to induce HDP expression in humans, rabbits, cattle, and chickens [24]–[27]. Several analogs of butyrate were further revealed to process a similar LL-37-inducing activity in human cells [28]. A comparison of the HDP-inducing activity among all saturated free fatty acids of different hydrocarbon chain lengths indicated that SCFAs are the most potent, with butyrate having the peak HDP-inducing activity in chicken cells [29]. Collectively, these studies raised the possibility of augmenting HDP synthesis as a novel antimicrobial approach for both humans and livestock animals [3].

However, not all animal species show a heightened production of HDPs in response to the aforementioned dietary compounds, and species-specific regulation of HDP synthesis exists. For example, vitamin D_3_ enhances HDP expression in humans, but not mice, rats or dogs [14]. Instead of promoting HDP production, butyrate and its derivatives are incapable of inducing mouse cathelicidin CRAMP in RAW264.7 macrophage cells (Zhang, G., et al., unpublished data) and even suppress CRAMP expression in mice following intraperitoneal administration [30]. Moreover, individual HDPs are known to be differentially regulated by dietary compounds. Among all 14 β-defensins and 4 cathelicidins in chickens, approximately a half number of HDPs are obviously induced in response to butyrate, although the magnitude of induction varies markedly among genes and cell types [26]. On the other hand, vitamin D_3_ was shown to induce LL-37 and β-defensin 2, but not several other defensins that were tested [14], [15].

In this study, we examined whether butyrate and its analogs have the ability to stimulate HDP gene expression in pigs. Three different porcine cell types including IPEC-J2 intestinal cells, 3D4/31 macrophage cells, and primary monocytes were used to evaluate possible regulation of a majority of known porcine HDPs in response to butyrate. Furthermore, a panel of saturated free fatty acids with 1–18 carbons as well as butyrate analogs was examined for their HDP-inducing activity in porcine IPEC-J2 cells. We revealed that a majority, but not all, porcine HDPs can be regulated by butyrate. We also identified several butyrate analogs with a strong capacity to induce HDP expression and great potential to be further explored as alternatives to antibiotics for animal agriculture.

## Materials and Methods

### Ethics Statement

This study was carried out in strict accordance with the recommendations in the Guide for the Care and Use of Laboratory Animals of the National Research Council. Animal procedures reported herein were approved by the Institutional Animal Care and Use Committee of Oklahoma State University.

### Chemicals

Sodium formate (C1), sodium acetate (C2), sodium propionate (C3), sodium butyrate (C4), valeric acid (C5), sodium hexanoate (C6), sodium *n*-octanoate (C8), sodium decanoate (C10), *trans*-cinnamyl butyrate, benzyl butyrate, glyceryl tributyrate (also known as tributyrin), 5-phenylvaleric acid, sodium 4-phenylbutyrate, hydrocinnamic acid (also known as 3-phenylpropanoic acid), 2-phenylacetic acid, and sodium benzoate were purchased from Sigma-Aldrich (St. Louis, MO), whereas sodium heptanoate (C7), sodium nonanoate (C9), sodium dodecanoate (C12), sodium tetradecanoate (C14), and sodium octadecanoate (C18) were from TCI America (Portland, OR). SCFAs (sodium formate, acetate, propionate, butyrate, and valeric acid), medium-chain fatty acids (sodium hexanoate, heptanoate, *n*-octanoate, nonanoate, and decanoate), sodium 4-phenylbutyrate, and sodium benzoate were dissolved in RPMI 1640 medium, whereas long-chain fatty acids (sodium dodecanoate, tetradecanoate, and octadecanoate) were dissolved in methanol, and benzyl butyrate, glyceryl tributyrate, *trans*-cinnamyl butyrate, and hydrocinnamic acid were dissolved in ethanol. On the other hand, 5-phenylvaleric acid and 2-phenylacetic acid were dissolved in dimethyl sulfoxide (DMSO).

### Cell Culture and Treatment

Porcine intestinal epithelial cell line, IPEC-J2 [31], was kindly provided by Dr. Bruce Schultz at Kansas State University (Manhattan, KS), whereas porcine lung alveolar macrophage cell line, 3D4/31 (ATCC #CRL-2844), was purchased from American Type Culture Collection (Manassas, VA). IPEC-J2 cells were cultured in 6-well plates at 1×10^6^ cells/well in DMEM/F12 medium supplemented with 10% fetal bovine serum (FBS), 100 µg/ml streptomycin, 100 U/ml penicillin, and 20 mM HEPES, pH 7.4. 3D4/31 cells were cultured in 6-well plates at 1×10^6^ cells/well in RPMI 1640 medium supplemented with 10% FBS, 100 µg/ml streptomycin, 100 U/ml penicillin, and 1 mM sodium pyruvate. After overnight growth, cells were treated in duplicate with various chemicals. All chemicals were first tested for their cytotoxicity. A broad range of sub-toxic concentrations of the chemicals were then used to determine their effect on HDP gene expression, and only an optimal HDP-inducing range of concentrations were presented for each compound. A series of time-course experiments were also conducted with sodium butyrate to determine the optimal duration of stimulation. All experiments were repeated at least 2–3 times. None of the solvents including methanol, ethanol, and DMSO showed any appreciable effect on HDP expression (data not shown).

### Porcine Primary Monocyte Isolation and Stimulation

Acid citrate dextrose-anticoagulated blood was freshly collected from the jugular vein of approximately 30- to 35-day-old pigs by venipuncture. Mononuclear cells were isolated by density gradient centrifugation using Histopaque 1077 (Sigma), followed by hypotonic lysis of the red blood cells, if needed. Mononuclear cells were then resuspended in RPMI 1640 medium supplemented with 10% FBS, 100 µg/ml streptomycin, 100 U/ml penicillin, and 20 mM HEPES and plated in 6-well plates at 1.5×10^7^ cells/well. After overnight at 37°C and 5% CO_2_, non-adherent cells were aspirated, and the plated were washed twice with PBS. Adherent cells, namely monocytes, were cultured in the same medium for mononuclear cells for 2 h prior to stimulation with different doses of sodium butyrate in duplicate for 24 h.

### Analysis of Porcine Gene Expression by Real Time RT-PCR

After stimulation, cells were lysed directly in RNAzol RT (Molecular Research Center, Cincinnati, OH). Total RNA was extracted according to the manufacturer’s instructions. RNA concentrations were measured by Nanodrop. The first-strand cDNA was synthesized using QuantiTect Reverse Transcription Kit (Qiagen). Real-time PCR was performed using QuantiTect SYBR Green PCR Kit (Qiagen) in 10-µl reactions with 1/20 (for GAPDH) or 1/10 (for HDPs) of the first-strand cDNA and gene-specific porcine HDP primers (Table 1) as we previously described [26], [29], [32], [33]. MyiQ Real-Time PCR System (Bio-Rad) was used, and the PCR program was set for initial denaturation at 95°C for 10 min, followed by 40 cycles of 94°C for 15 sec, 55°C for 20 sec, and 72°C for 30 sec. The specificity of PCR amplification was confirmed by the melt curve analysis. Relative gene expression was calculated according to the ΔΔCt method using porcine glyceraldehyde-3-phosphate dehydrogenase (GAPDH) as the reference gene as described [26], [29], [32], [33].

**Table 1:** Primer sequences of porcine host defense peptides for real time PCR.

Gene	Forward primer	Reverse primer	Product Size (bp)
pBD1	TTCCTCCTCATGGTCCTGTT	AGGTGCCGATCTGTTTCATC	130
pBD2	TGTCTGCCTCCTCTCTTCC	AACAGGTCCCTTCAATCCTG	149
pBD3	CCTTCTCTTTGCCTTGCTCTT	GCCACTCACAGAACAGCTACC	163
pBD4	GTCACAATTTTACCAGCCAGAA	GCAATCCTAATTTCACCCTCA	122
pBD123	GATTTTGACTGTTGCCTTGCT	TGAGGATTCAGGTTTTGATCG	176
pBD125	CATGAATCTCCTGCTGACCTT	TCATCACTGCTTAGGGGAATG	186
pBD129	TCCTATCTTTGCCAGCCTCA	ATCTCCCCAAACCCATTACAC	86
pEP2C	CCCTGGACAAGAAACAAACAA	TGACATCTGCCTTCACTTCTC	234
PG1-5	GTAGGTTCTGCGTCTGTGTCG	CAAATCCTTCACCGTCTACCA	166
PF1-2	CCTACGAAGACCACGGTTGC	CAAATCCTTCACCGTCTACCA	285
PR-39	AGCAGTCCTCGGAAGCTAATC	GTCATTGGATGGGTTCAAGGT	215
PMAP-23	GGATTATAGACCTGCTGTGGA	AGAACTCTTCCCTGTGTCTTG	118
PMAP-36	CTTGAACCCATCCAATGACC	TCAAAACCTTCCCGATCTTC	119
PMAP-37	CTACTTAGCCGACTGCGTGAT	GATAGCCTGAATCTTAGGACTG	125
IL-1β	GTGTTCTGCATGAGCTTTGTG	GGCTTTCCTTAGGGAGAGAGG	347
GAPDH	ATCACTGCCACCCAGAAGAC	AGCCCCAGCATCAAAGGTAG	357

### Statistics Analysis

Statistical analyses were performed using GraphPad PRISM 5 (GraphPad Software, La Jolla, CA). All pairwise comparisons were examined using unpaired Student’s two-tailed *t*-test. Differences were considered significant when P<0.05.

## Results

### Sodium Butyrate Stimulates HDP Gene Expression in Different Porcine Cell Types

Sodium butyrate is a strong inducer of HDP genes in humans, rabbits, cattle, and chickens [24]–[27], but not in mice [30]. To test whether butyrate could increase the HDP gene expression in pigs, we performed both dose-response and time-course experiments in porcine IPEC-J2 intestinal epithelial cells and then analyzed the expressions of multiple porcine HDP genes by real-time RT-PCR. The genes that were examined included all 11 known cathelicidins (PG1-5, PF1-2, PR-39, PMAP-23, PMAP-36, and PMAP-37) and seven β-defensins with adequate expressions in epithelial tissues (pBD1, pBD2, pBD3, pBD4, pBD123, pBD125, pBD129, and pEP2C) [12], [13]. The β-defensins that are lowly expressed in most tissues (e.g., pBD104 and pBD114) as well as those that are highly specific to male reproductive tissues (e.g., pBD108) were excluded from the current study. It is noted that, because of a high sequence similarity among five protegrins, the primers were designed to simultaneously target all five members (PG1-5), rather than individual ones. The same is true for the primers for two prophenins (PF1-2).

**Figure 1:**
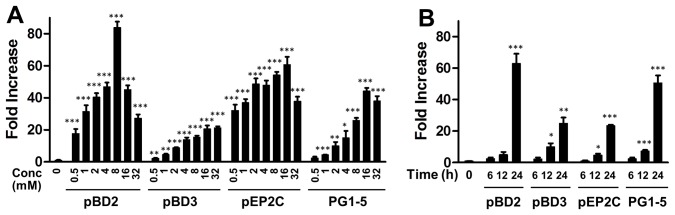
Butyrate-induced expression of pBD2, pBD3, pEP2C, and PG1-5 in porcine IPEC-J2 intestinal epithelial cells. Cells were incubated in duplicate with indicated concentrations of butyrate for 24 h (A) or 8 mM of butyrate for 6, 12, and 24 h (B). Gene expression was analyzed by real-time RT-PCR. The relative fold changes over the unstimulated control were calculated with the ΔΔCt method using porcine glyceraldehyde-3-phosphate dehydrogenase (GAPDH) gene for normalization. Data were means ± standard error of a representative of two independent experiments. *P<0.05, **P<0.01, and ***P<0.001 (in comparison with solvent controls by unpaired Student’s *t*-test).

Our results indicated that a 24-h treatment with sodium butyrate markedly increased pBD2 expression in a dose-dependent manner in IPEC-J2 cells, peaking at 8 mM (Fig. 1A). Similarly, the expression levels of pBD3, pEP2C, and PG1-5 were also dose-dependently induced by butyrate in IPEC-J2 cells, with the maximal response occurring at 16 mM (Fig. 1A). However, the magnitude of induction varied obviously among the four genes, with pBD2 showing an approximately 85-fold increase, and pBD3, pEP2C, and PG1-5 showing 20-, 60-, and 45-fold induction at the peak response (Fig. 1A). An obvious time-dependent induction of pBD2, pBD3, pEP2C, and PG1-5 was also observed in IPEC-J2 cells following 8 mM butyrate treatment (Fig. 1B). A maximal response occurred at 24 h, whereas 6 and 12 h treatments only showed a marginal effect, which is consistent with earlier observations in human and chicken cells [24], [26].

PMAP-23, PMAP-37, and PR-39 were also evidently induced in IPEC-J2 cells in response to butyrate treatment; however, because of our inability to detect the basal expression levels of these genes in unstimulated cells, the data were not included in the analysis. It is noteworthy that pBD1 expression was readily detected, but remained largely unaltered in IPEC-J2 cells following butyrate treatment (data not shown). We failed to detect the expressions of pBD4, pBD123, pBD125, pBD129, PF1-2, and PMAP36 in IPEC-J2 cells before and after butyrate treatment (data not shown).

**Figure 2:**
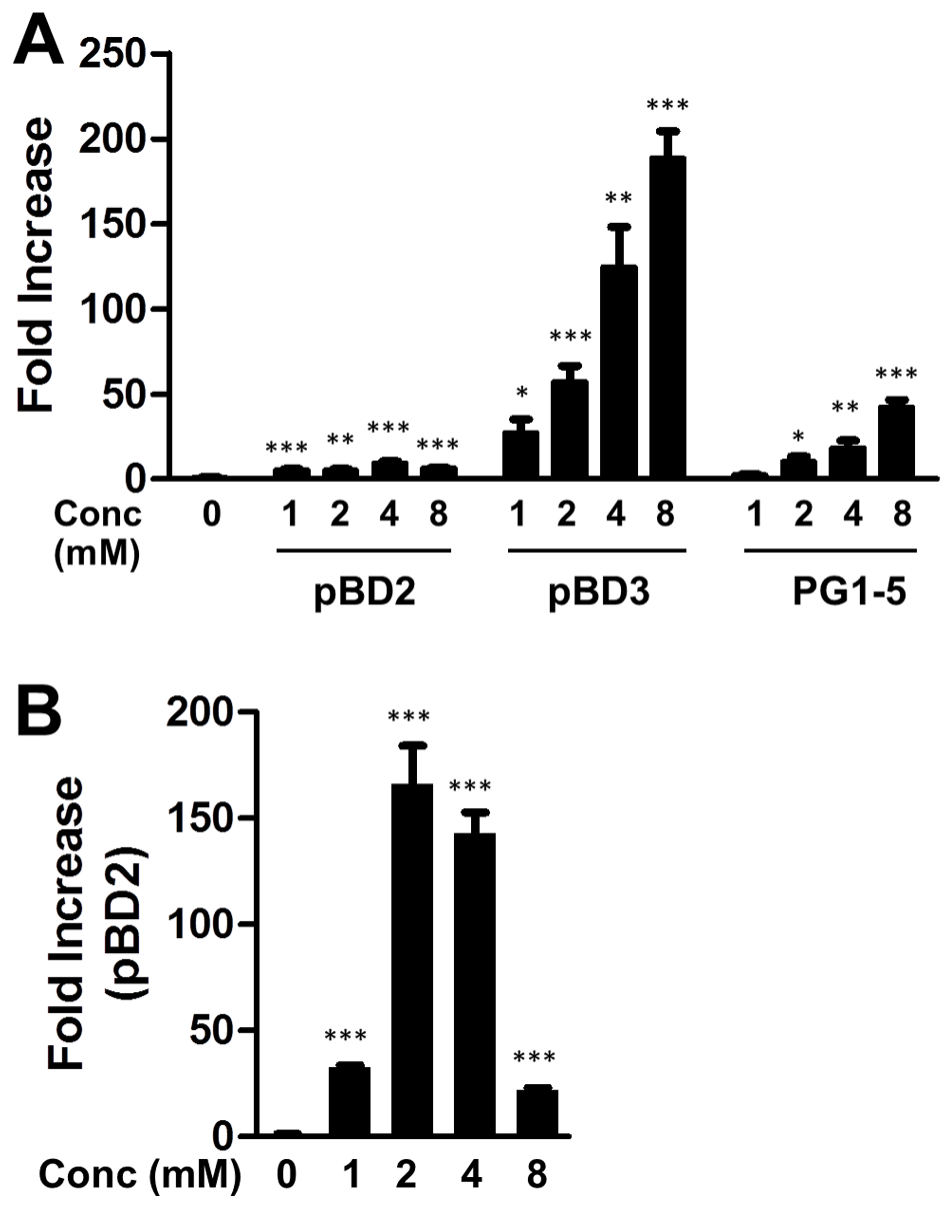
Butyrate-induced expression of pBD2, pBD3, and PG1-5 in porcine 3D4/31 macrophage cells (A) and pBD2 in primary monocytes (B). Cells were incubated in duplicate with indicated concentrations of butyrate for 24 h. Gene expression was analyzed by real-time RT-PCR. The relative fold changes over the unstimulated control were calculated with the ΔΔCt method using porcine glyceraldehyde-3-phosphate dehydrogenase (GAPDH) gene for normalization. Data were means ± standard error of a representative of 2–3 independent experiments. *P<0.05, **P<0.01, and ***P<0.001 (in comparison with solvent controls by unpaired Student’s *t*-test).

To further examine whether butyrate is capable of stimulating the porcine HDP expression in other cell types, porcine 3D4/31lung alveolar macrophage cells were treated with different doses of butyrate for 24 h. A dose-dependent augmentation of pBD2, pBD3, and PG1-5 was observed (Fig. 2A), although the induction patterns of the three genes were in sharp contrast with those in IPEC-J2 cells (Fig. 1A), where pBD2 was more markedly upregulated by butyrate than pBD3. In contrast, pBD3 showed much more dramatic change in 3D4/31 cells, with a nearly 190-fold increase following 8 mM butyrate treatment, whereas pBD2 was only marginally induced by less than 10-fold at any dose applied. PG1-5 showed an intermediate 42-fold increase in 3D4/31 cells in response to 8 mM butyrate (Fig. 2A). Like in IPEC-J2 cells, PMAP-37 was obviously induced in 3D4/31 cells by butyrate; however, we could not calculate relative fold induction because of an inability to detect its expression in untreated cells. The expression of pBD1 remained unchanged in 3D4/31 cells in response to butyrate (data not shown). Other HDP genes such as pBD4, PMAP-36, and PR-39 could not be detected in 3D4/31 cells before or after butyrate treatment.

Butyrate-induced expression of porcine HDPs was further confirmed in primary monocytes. A 24-h treatment of primary monocytes with sodium butyrate dose-dependently enhanced the mRNA expression of pBD2, with 2 or 4 mM butyrate giving an approximately 150-fold increase, in comparison with the control (Fig. 2B). However, we could not reliably detect the expression of pBD3, PG1-5, and multiple other HDP genes in porcine primary monocytes.

Butyrate is known to be anti-inflammatory and has been used to treat inflammatory bowel diseases [34]. To examine the influence of butyrate on the expression of a major proinflammatory cytokine, interleukin (IL)-1β, real-time RT-PCR was performed on both IPEC-J2 cells and primary monocytes. IL-1β was not detectable in IPEC-J2 cells either before or 6, 12 or 24 h after 8 mM butyrate treatment, whereas a minimal 2.5-fold increase was seen in primary monocytes 24 h after stimulation with 8 mM butyrate (data not shown), suggesting that butyrate has a minimal impact on the inflammatory response in porcine cells, which is reminiscent of our earlier observations in chickens, where IL-1β and IL12p40 expressions were largely unaffected by butyrate in HD11 macrophage cells [26]. Because of the fact that SCFAs are naturally produced in the intestinal tract and quickly metabolized and our expectation that SCFAs mainly work locally in the digestive tract rather than systemically in the circulation, IPEC-J2 cells were chosen for all subsequent studies.

### Fatty Acid Chain Length is Correlated with the HDP-inducing Activity of Free Fatty Acids

**Figure 3:**
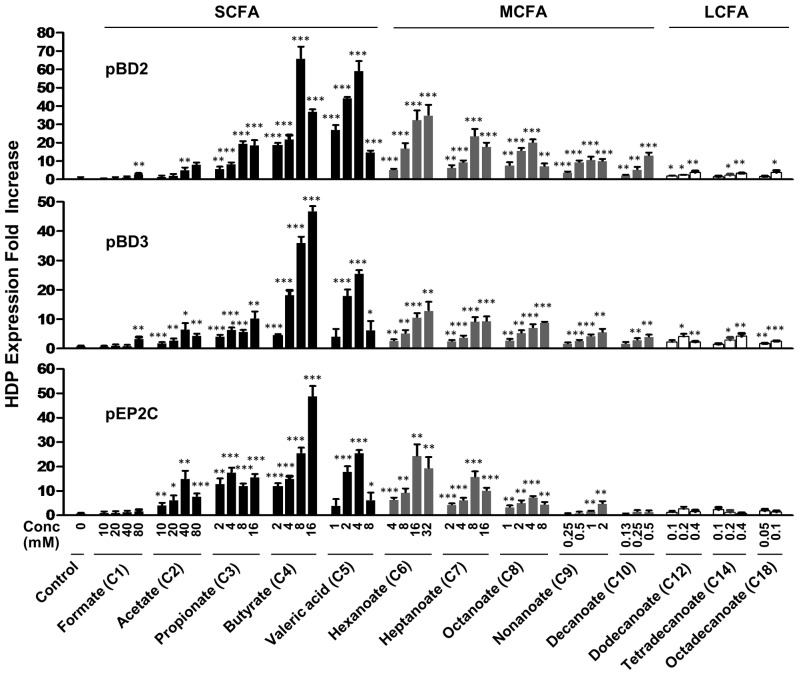
Regulation of pBD2, pBD3, and pEP2C expression by saturated free fatty acids. Porcine IPEC-J2 cells were incubated in duplicate with indicated concentrations of saturated free fatty acids for 24 h. Gene expression was analyzed by real-time RT-PCR. The relative fold changes over the unstimulated control were calculated with the ΔΔCt method using porcine glyceraldehyde-3-phosphate dehydrogenase (GAPDH) gene for normalization. Data were means ± standard error of a representative of 2–3 independent experiments. Abbreviations: SCFA, short-chain fatty acids; MCFA, medium-chain fatty acids; LCFA, long-chain fatty acids. *P<0.05, **P<0.01, and ***P<0.001 (in comparison with solvent controls by unpaired Student’s *t*-test).

We previously showed that butyrate is the most potent in inducing HDP gene expression among all free fatty acids of different hydrocarbon chain lengths in chickens [29]. To compare the relative HDP-inducing efficacy of saturated free fatty acids in pigs, IPEC-J2 cells were treated first with a broad range of doses of different fatty acids with the chain length varying from C1 to C18 for 24 h, and the cytotoxicity was examined as we previously described [35]–[37]. Generally, fatty acids showed an enhanced toxicity as the aliphatic chain length increased (data not shown). Different doses of the subtoxic doses of fatty acids were then evaluated for their effect on the induction of pBD2, pBD3, and pEP2C by real-time RT-PCR. Obviously, each free fatty acid showed a largely dose-dependent induction of all three HDP genes tested (Fig. 3).

Furthermore, there was a clear correlation between the chain length and the HDP-inducing efficacy among all free fatty acids. For the fatty acids with 4 carbons or less, the HDP-inducing activity was gradually enhanced as the chain length increased; however, a length-dependent decrease in the HDP-inducing activity was observed for the free fatty acids with more than 4 carbons (Fig. 3). The longer the fatty acid, the weaker is its ability to induce HDP expression. Fatty acids with 3–8 carbons possessed a strong HDP-inducing activity with an obvious induction of pBD2, pBD3, and pEP2C. The peak response occurred with sodium butyrate (C4), whereas formate (C1) and long-chain fatty acids (LCFAs) (≥ C12) showed only a marginal effect on regulating HDP gene expression (Fig. 3). Collectively, these results are consistent with our earlier observation in chicken HD11 macrophage cells and primary monocytes, where butyrate exhibited the most potent HDP-inducing activity [29].

### Butyrate Analogs Differentially Enhance HDP Expression

**Figure 4:**
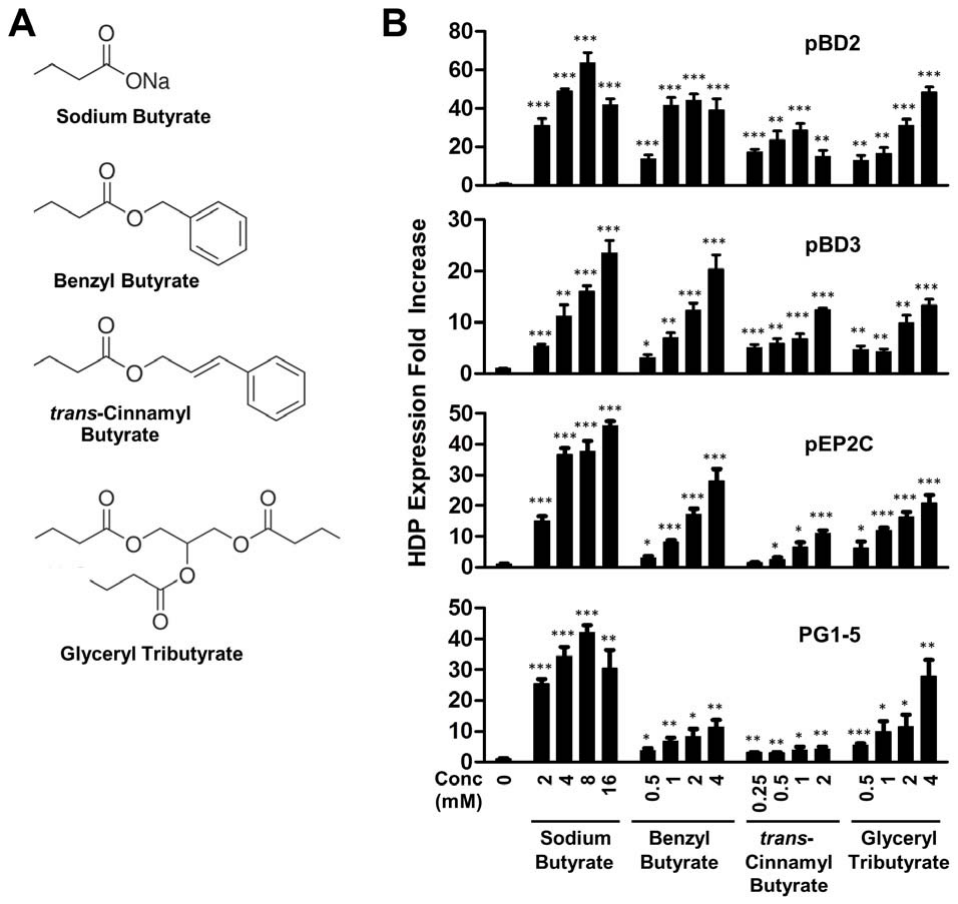
Regulation of pBD2, pBD3, pEP2C, and PG1-5 expression by butyrate and its analogs. Porcine IPEC-J2 cells were inbubated in duplicate with indicated concentrations of each compound (A) for 24 h. Gene expression was analyzed by real-time RT-PCR. The relative fold changes (B) over the unstimulated control were calculated with the ΔΔCt method using porcine glyceraldehyde-3-phosphate dehydrogenase (GAPDH) gene for normalization. Data were means ± standard error of a representative of 2–3 independent experiments. *P<0.05, **P<0.01, and ***P<0.001 (in comparison with solvent controls by unpaired Student’s *t*-test).

Because of an unpleasant smell and a quick metabolic rate, butyrate is not a particularly attractive compound for in vivo applications [3]. Odorless and/or structurally rigid analogs of butyrate have stronger potential. Several butyrate analogs including 4-phenylbutyrate and α-methylhydrocinnamate were recently shown to be comparable to butyrate in HDP induction in human cells [28]. To identify the butyrate analogs with a potent activity in stimulating HDP expression in pigs, IPEC-J2 cells were treated for 24 h with different doses of benzyl butyrate, glyceryl tributyrate, and *trans*-cinnamyl butyrate (see their chemical structures in Fig. 4A). The expressions of pBD2, pBD3, pEP2C, and PG1-5 were then analyzed by real-time RT-PCR. All three analogs triggered an evident dose-dependent increase in the expressions of all four HDP genes (Fig. 4B).

At the optimal HDP-inducing dose, glyceryl tributyrate, an odorless esterified derivative consisting of three butyric acids, showed a comparable potency to sodium butyrate in inducing all four HDPs, with a less than 2-fold difference in the peak response (Fig. 4B). Benzyl butyrate, a common ingredient of fragrances with a fruity smell, was also approaching to the efficiency of butyrate in regulating pBD2, pBD3, and pEP2C at 4 mM. However, benzyl butyrate was 4-fold less efficient in stimulating PG1-5 expression than butyrate at the optimal dose (4 mM). *Trans*-cinnamyl butyrate was obviously weaker in inducing all four HDPs than butyrate. It is particularly the case in the induction of PG1-5. An approximately 4-fold maximal increase in the PG1-5 expression was observed with *trans*-cinnamyl butyrate at 2 mM, whereas a 42-fold peak increase was seen with butyrate at 8 mM (Fig. 4B). Even at 2 mM, butyrate triggered a 25-fold increase in the expression of PG1-5, suggesting that butyrate analogs vary greatly in their ability to regulate HDP gene expression.

### Impact of the Aromatic Ring on SCFA-induced HDP Expression

**Figure 5:**
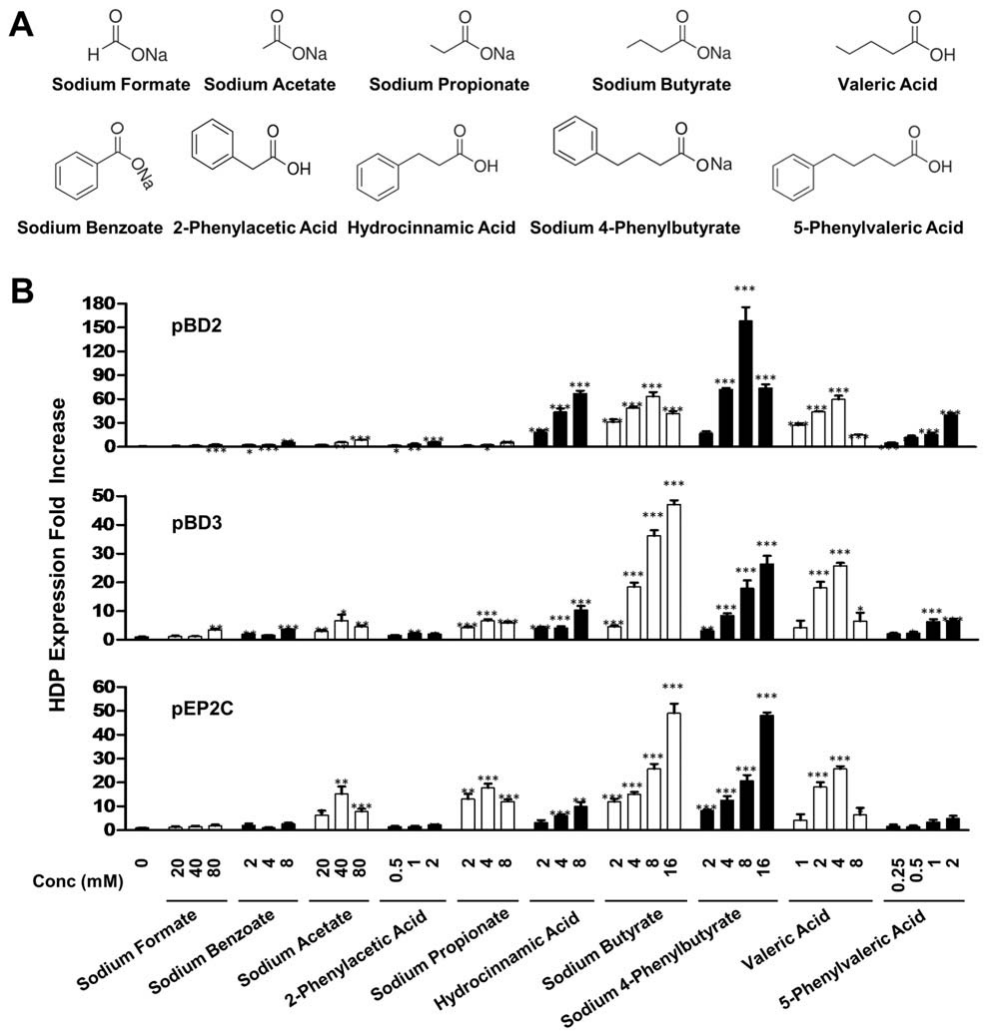
Regulation of pBD2, pBD3, and pEP2C expression by short-chain fatty acids and their phenyl derivatives. Porcine IPEC-J2 cells were inbubated in duplicate with indicated concentrations of each compound (A) for 24 h. HDP gene expression was analyzed by real-time PCR. The relative fold changes (B) over the unstimulated control were calculated with the ΔΔCt method using porcine glyceraldehyde-3-phosphate dehydrogenase (GAPDH) gene for normalization. Data were means ± standard error of a representative of 2–3 independent experiments. *P<0.05, **P<0.01, and ***P<0.001 (in comparison with solvent controls by unpaired Student’s *t*-test).

Sodium 4-phenylbutyrate is more efficient than butyrate in inducing HDP expression in human lung epithelial cells [28], but comparable to butyrate in several chicken cell lines (data not shown). To examine the impact of the aromatic ring on SCFA-induced HDP expression in pigs, IPEC-J2 cells were stimulated for 24 h with different doses of free SCFAs (C1-C5) or their corresponding analogs with a phenyl group attached at the end of the aliphatic chain (see their structures in Fig. 5A). It became apparent that the presence of a phenyl group substantially increased the toxicity of SCFAs to IPEC-J2 cells, except for butyrate and propionate, which showed a similar toxicity to 4-phenylbutyrate and hydrocinnamic acid, respectively (data not shown). Subtoxic doses of phenyl derivatives of SCFAs were then used to identify the optimal concentration range to induce pBD2, pBD3, pEP2C, and PG1-5 by real-time RT-PCR. Similar to free SCFAs, all phenyl derivatives showed a chain length-dependent HDP expression, with 4-phenylbutyrate (C4) being the most potent. For those derivatives consisting of an aliphatic chain length of 4 carbons or less, the HDP-inducing activity decreased as the chain shortened (compare among solid bars in Fig. 5B). On the other hand, the opposite was true between 4-phenylbutyrate and 5-phenylvaleric acid (C5). Overall, 4-phenylbutyrate, hydrocinnamic acid (C3), and 5-phenylvaleric acid showed an obvious induction of all four porcine HDPs examined, whereas 2-phenylacetic acid (C2) and sodium benzoate (C1) exhibited largely a marginal effect on HDP expression.

When compared between a free SCFA and its corresponding phenyl derivative, the influence of the phenyl group on the HDP-inducing activity varied markedly among individual pairs. For example, at the identical concentrations, 4-phenylbutyrate was more efficient than butyrate in inducing pBD2 expression, with more than 2-fold in difference in the peak response. In contrast, butyrate was generally 2-fold more efficient than 4-phenylbutyrate in pBD3 induction at each concentration tested. On the other hand, 4-phenylbutyrate and butyrate were largely comparable in their efficacy in inducing the expressions of pEP2C (Fig. 5B) and PG1-5 (data not shown). In the case of valeric acid and 5-phenylvaleric acid, the former was invariably more potent in inducing all four HDPs than the latter. The same trend held true between acetate and 2-phenylacetic acid, with the free fatty acid showing an augmented HDP expression. Between propionate and hydrocinnamic acid, the presence of a phenyl group obviously enhanced pBD2 expression, but suppressed pEP2C expression with a minimal impact on pBD3 expression. Collectively, the phenyl group had a mixed effect on HDP induction that varied obviously among individual genes and SCFAs.

## Discussion

Although many dietary compounds possess the HDP-inducing activity, species-specific differences clearly exist at least in the case of vitamin D_3_ and butyrate [14], [30]. It is thus necessary to examine the ability of individual compounds to induce HDPs in each species. In this study, we have confirmed that butyrate is a strong inducer of multiple β-defensin and cathelicidin genes in different porcine cell types. It is known that LL-37 mRNA and protein expressions are concurrently augmented in humans and rabbits following butyrate administration [25], [38], and that butyrate-induced HDP mRNA expression is associated with an enhanced antibacterial activity of monocytes and bacterial clearance in chickens [26]. It is still important to confirm the up-regulation of porcine HDPs at the protein levels, particularly in vivo, and the subsequent effect on disease resistance and pathogen reduction in pigs in the future.

In addition to species-specific regulation of HDPs by butyrate, gene- and cell-type specific regulation patterns are also evident. First, not all HDP genes are regulated by butyrate. Among all porcine HDPs that were analyzed, pBD1 is clearly un-inducible in any cell type. Secondly, for those HDP genes that are induced by butyrate, each is differentially regulated, with a different magnitude of induction in any given cell type. For example, in 3D4/31 macrophages, pBD3 was induced by 188-fold, whereas pBD2 and PG1-5 were increased only by 6.6- and 42-fold, respectively, in response to 8 mM butyrate (Fig. 2A). Thirdly, the same HDP gene may be regulated differently in different cell types. The expression of pBD2 was enhanced by 84-fold in IPEC-J2 cells following 8 mM butyrate treatment (Fig. 1A), yet a minimum 6.6-fold induction was seen in 3D4/31 macrophages (Fig. 2A). Such a gene- and cell-type specific regulation of porcine HDPs was also clearly demonstrated in humans and chickens [26], [39]. It is thus important to verify the HDP regulation in different cell types.

Butyrate is the main SCFA derived from fermentation of undigested dietary starches and fiber in the large intestine of animals [40], [41]. Together with other SCFAs, butyrate exerts pleiotropic effects on the pathophysiology of intestinal functions. The molecular mechanisms by which butyrate induces LL-37 gene expression have been investigated in human cells. Butyrate-mediated LL-37 induction has been found to be associated with hyperacetylation of core histones H3/H4 and a non-histone protein, high mobility group nucleosomal binding domain 2 (HMGN2) in intestinal epithelial cells [42], which could in turn cause DNA to be loosened up and a set of genes including LL-37 to be transactivated. In line with this, several histone deacetylase inhibitors such as trichostatin A and sulforaphane are indeed capable of inducing HDP expression in human cells [42], [43]. Additionally, mitogen-activated protein kinases, transforming growth factor β type 1-receptor kinase, and vitamin D receptor are all involved in butyrate-induced LL-37 expression in human colonic epithelial cells [44]. It is important to confirm the mechanisms of butyrate-induced porcine HDP expression in the future. On the other hand, vitamin D_3_-mediated LL-37 induction primarily relies on translocation and binding of vitamin D receptor to the vitamin D response element on the gene promoter [14], [15] and histone hyperacetylation is beneficial as vitamin D_3_ and histone deacetylase inhibitors such as butyrate synergizes in augmenting LL-37 expression [45].

A comparison of the HDP-inducing potency among saturated free fatty acids of various chain lengths revealed a “bell-shaped” curve in porcine IPEC-J2 intestinal epithelial cells, where fatty acids comprised of 3–8 carbons are highly potent, with butyrate being the most efficacious (Fig. 3). The results are reminiscent of our earlier data in chickens, where SCFAs, and butyrate in particular, showed the most dramatic HDP induction in HD11 macrophages and primary monocytes [29]. However, although LCFAs (≥12 carbons) exhibit a negligible effect on HDP induction in porcine intestinal epithelial cells, a low but obvious HDP induction was observed in chicken primary monocytes [29]. On the other hand, stimulation of human sebocytes with LCFAs such as lauric acid (C12:0), palmitic acid (C16:0), and oleic acid (C18:1, cis-9) drastically induced human β-defensin 2 expression, but not β-defensin 1, β-defensin 3 or cathelicidin LL-37 [46]. The results collectively implied the magnitude of HDP expression induced by different fatty acids is species-, cell type- and/or gene-dependent. In comparison with saturated LCFAs, unsaturated fatty acids appear to be stronger in inducing HDP expression in chicken monocytes [29]. It will be interesting to investigate the impact of the saturation status on the HDP-inducing activity of fatty acids in pigs.

Although 4-phenylbutyrate is more potent in LL-37 induction than butyrate in human VA10 bronchial epithelial cells [28], it is only true with pBD2 in porcine IPEC-J2 intestinal epithelial cells. In fact, a comparable efficacy in inducing three other porcine HDPs, namely pBD3, pEP2C, and PG1-5, was observed between 4-phenylbutyrate and butyrate, indicating a gene-specific response (Fig. 5B). Moreover, there is no clear pattern on the impact of the phenyl group on the HDP-inducing activity among free SCFAs. Phenyl SCFA derivatives showed varied efficacies in HDP induction as compared with the corresponding free SCFAs even for the same HDP gene (Fig. 5B). We also revealed that, in addition to 4-phenylbutyrate, glyceryl tributyrate and benzyl butyrate showed a comparable efficacy in induction of all four porcine HDPs (Fig. 4B). The ability of these compounds to enhance HDP expression in porcine intestinal epithelial cells is particularly beneficial for strengthening the mucosal antimicrobial barrier, especially in newborn and weaning piglets, which have an immature immune system, are susceptible to infections, and often suffer from diarrhea and growth retardation if infected [47].

Sodium 4-phenylbutyrate is an odorless butyrate analog that has been approved for treatment of urea cycle disorders and is under investigation for multiple other clinical indications [48]. Glyceryl tributyrate (also known as tributyrin) is another odorless butyrate prodrug with more favorable pharmacokinetic properties than butyrate [49]. On the other hand, benzyl butyrate is a commonly used ingredient in fragrances with a pleasant plum smell and extensive animal toxicity data [50]. In conclusion, identification of these butyrate analogs with favorable odors, known safety features, and/or improved pharmacokinetic properties will expedite their application as non-antibiotic, immune boosting dietary additives for infectious disease control and prevention in pigs and perhaps in other animal species including humans as well.

## References

[1] McDermott PF, Zhao S, Wagner DD, Simjee S, Walker RD, et al (2002) The food safety perspective of antibiotic resistance. Anim Biotechnol 13: 71–84. 12212946 10.1081/ABIO-120005771

[2] Smith DL, Dushoff J, Morris JG (2005) Agricultural antibiotics and human health. PLoS Med 2: e232. 15984910 10.1371/journal.pmed.0020232 PMC1167557

[3] van der Does AM, Bergman P, Agerberth B, Lindbom L (2012) Induction of the human cathelicidin LL-37 as a novel treatment against bacterial infections. J Leukoc Biol 92: 735–742. 22701042 10.1189/jlb.0412178

[4] Pasupuleti M, Schmidtchen A, Malmsten M (2012) Antimicrobial peptides: key components of the innate immune system. Crit Rev Biotechnol 32: 143–171. 22074402 10.3109/07388551.2011.594423

[5] Takahashi D, Shukla SK, Prakash O, Zhang G (2010) Structural determinants of host defense peptides for antimicrobial activity and target cell selectivity. Biochimie 92: 1236–1241. 20188791 10.1016/j.biochi.2010.02.023

[6] Choi KY, Chow LN, Mookherjee N (2012) Cationic host defence peptides: multifaceted role in immune modulation and inflammation. J Innate Immun 4: 361–370. 22739631 10.1159/000336630 PMC6741489

[7] Pulido D, Nogues MV, Boix E, Torrent M (2012) Lipopolysaccharide neutralization by antimicrobial peptides: a gambit in the innate host defense strategy. J Innate Immun 4: 327–336. 22441679 10.1159/000336713 PMC6741597

[8] Selsted ME, Ouellette AJ (2005) Mammalian defensins in the antimicrobial immune response. Nat Immunol 6: 551–557. 15908936 10.1038/ni1206

[9] Zanetti M (2004) Cathelicidins, multifunctional peptides of the innate immunity. J Leukoc Biol 75: 39–48. 12960280 10.1189/jlb.0403147

[10] Sang Y, Blecha F (2009) Porcine host defense peptides: expanding repertoire and functions. Dev Comp Immunol 33: 334–343. 18579204 10.1016/j.dci.2008.05.006

[11] Zhang G, Ross CR, Blecha F (2000) Porcine antimicrobial peptides: new prospects for ancient molecules of host defense. Vet Res 31: 277–296. 10863946 10.1051/vetres:2000121

[12] Zhang G, Wu H, Shi J, Ganz T, Ross CR, et al (1998) Molecular cloning and tissue expression of porcine beta-defensin-1. FEBS Lett 424: 37–40. 9537511 10.1016/s0014-5793(98)00134-3

[13] Sang Y, Patil AA, Zhang G, Ross CR, Blecha F (2006) Bioinformatic and expression analysis of novel porcine beta-defensins. Mamm Genome 17: 332–339. 16596454 10.1007/s00335-005-0158-0

[14] Gombart AF, Borregaard N, Koeffler HP (2005) Human cathelicidin antimicrobial peptide (CAMP) gene is a direct target of the vitamin D receptor and is strongly up-regulated in myeloid cells by 1,25-dihydroxyvitamin D3. FASEB J 19: 1067–1077. 15985530 10.1096/fj.04-3284com

[15] Wang TT, Nestel FP, Bourdeau V, Nagai Y, Wang Q, et al (2004) Cutting edge: 1,25-dihydroxyvitamin D3 is a direct inducer of antimicrobial peptide gene expression. J Immunol 173: 2909–2912. 15322146 10.4049/jimmunol.173.5.2909

[16] Cederlund A, Kai-Larsen Y, Printz G, Yoshio H, Alvelius G, et al (2013) Lactose in human breast milk an inducer of innate immunity with implications for a role in intestinal homeostasis. PLoS One 8: e53876. 23326523 10.1371/journal.pone.0053876 PMC3542196

[17] Sherman H, Chapnik N, Froy O (2006) Albumin and amino acids upregulate the expression of human beta-defensin 1. Mol Immunol 43: 1617–1623. 16263169 10.1016/j.molimm.2005.09.013

[18] Mao XB, Qi S, Yu B, Huang ZQ, Chen H, et al (2012) Dietary L-arginine supplementation enhances porcine beta-defensins gene expression in some tissues of weaned pigs. Livestock Science 148: 103–108.

[19] Fehlbaum P, Rao M, Zasloff M, Anderson GM (2000) An essential amino acid induces epithelial beta -defensin expression. Proc Natl Acad Sci USA 97: 12723–12728. 11058160 10.1073/pnas.220424597 PMC18831

[20] Mao X, Qi S, Yu B, He J, Yu J, et al (2013) Zn(2+) and L-isoleucine induce the expressions of porcine beta-defensins in IPEC-J2 cells. Mol Biol Rep 40: 1547–1552. 23065293 10.1007/s11033-012-2200-0

[21] Talukder P, Satho T, Irie K, Sharmin T, Hamady D, et al (2011) Trace metal zinc stimulates secretion of antimicrobial peptide LL-37 from Caco-2 cells through ERK and p38 MAP kinase. Int Immunopharmacol 11: 141–144. 21035435 10.1016/j.intimp.2010.10.010

[22] Zhang JH, Deng J, Li YF, Yang Q (2011) The effect of Lactobacillus on the expression of porcine beta-defensin-2 in the digestive tract of piglets. Livestock Science 138: 259–265.

[23] Schlee M, Harder J, Koten B, Stange EF, Wehkamp J, et al (2008) Probiotic lactobacilli and VSL#3 induce enterocyte beta-defensin 2. Clin Exp Immunol 151: 528–535. 18190603 10.1111/j.1365-2249.2007.03587.x PMC2276967

[24] Schauber J, Svanholm C, Termen S, Iffland K, Menzel T, et al (2003) Expression of the cathelicidin LL-37 is modulated by short chain fatty acids in colonocytes: relevance of signalling pathways. Gut 52: 735–741. 12692061 10.1136/gut.52.5.735 PMC1773650

[25] Raqib R, Sarker P, Bergman P, Ara G, Lindh M, et al (2006) Improved outcome in shigellosis associated with butyrate induction of an endogenous peptide antibiotic. Proc Natl Acad Sci U S A 103: 9178–9183. 16740661 10.1073/pnas.0602888103 PMC1482586

[26] Sunkara LT, Achanta M, Schreiber NB, Bommineni YR, Dai G, et al (2011) Butyrate enhances disease resistance of chickens by inducing antimicrobial host defense peptide gene expression. PLoS One 6: e27225. 22073293 10.1371/journal.pone.0027225 PMC3208584

[27] Ochoa-Zarzosa A, Villarreal-Fernandez E, Cano-Camacho H, Lopez-Meza JE (2009) Sodium butyrate inhibits Staphylococcus aureus internalization in bovine mammary epithelial cells and induces the expression of antimicrobial peptide genes. Microb Pathog 47: 1–7. 19393738 10.1016/j.micpath.2009.04.006

[28] Steinmann J, Halldorsson S, Agerberth B, Gudmundsson GH (2009) Phenylbutyrate induces antimicrobial peptide expression. Antimicrob Agents Chemother 53: 5127–5133. 19770273 10.1128/AAC.00818-09 PMC2786349

[29] Sunkara LT, Jiang W, Zhang G (2012) Modulation of antimicrobial host defense peptide gene expression by free fatty acids. PLoS One 7: e49558. 23166711 10.1371/journal.pone.0049558 PMC3499459

[30] Huehnken C (2009) Gene expression in mammalian innate immunity: 4-PBA and ST7 reduce CRAMP gene expression in murine lung epithelial cells. Thesis, University of Ireland, Reykjavik, Iceland. Available: http://hdl.handle.net/1946/3062. Accessed: 1 Apr 2013.

[31] Schierack P, Nordhoff M, Pollmann M, Weyrauch KD, Amasheh S, et al (2006) Characterization of a porcine intestinal epithelial cell line for in vitro studies of microbial pathogenesis in swine. Histochem Cell Biol 125: 293–305. 16215741 10.1007/s00418-005-0067-z

[32] Xiao Y, Herrera AI, Bommineni YR, Soulages JL, Prakash O, et al (2009) The Central Kink Region of Fowlicidin-2, an alpha-Helical Host Defense Peptide, Is Critically Involved in Bacterial Killing and Endotoxin Neutralization. J Innate Immun 1: 268–280. 20375584 10.1159/000174822 PMC7312843

[33] Achanta M, Sunkara LT, Dai G, Bommineni YR, Jiang W, et al (2012) Tissue expression and developmental regulation of chicken cathelicidin antimicrobial peptides. J Anim Sci Biotechnol 3: 15. 22958518 10.1186/2049-1891-3-15 PMC3436658

[34] Canani RB, Costanzo MD, Leone L, Pedata M, Meli R, et al (2011) Potential beneficial effects of butyrate in intestinal and extraintestinal diseases. World J Gastroenterol 17: 1519–1528. 21472114 10.3748/wjg.v17.i12.1519 PMC3070119

[35] Bommineni YR, Achanta M, Alexander J, Sunkara LT, Ritchey JW, et al (2010) A fowlicidin-1 analog protects mice from lethal infections induced by methicillin-resistant Staphylococcus aureus. Peptides 31: 1225–1230. 20381563 10.1016/j.peptides.2010.03.037

[36] Bommineni YR, Dai H, Gong YX, Soulages JL, Fernando SC, et al (2007) Fowlicidin-3 is an alpha-helical cationic host defense peptide with potent antibacterial and lipopolysaccharide-neutralizing activities. FEBS J 274: 418–428. 17229147 10.1111/j.1742-4658.2006.05589.x

[37] Xiao Y, Dai H, Bommineni YR, Soulages JL, Gong YX, et al (2006) Structure-activity relationships of fowlicidin-1, a cathelicidin antimicrobial peptide in chicken. FEBS J 273: 2581–2593. 16817888 10.1111/j.1742-4658.2006.05261.x

[38] Raqib R, Sarker P, Mily A, Alam NH, Arifuzzaman AS, et al (2012) Efficacy of sodium butyrate adjunct therapy in shigellosis: a randomized, double-blind, placebo-controlled clinical trial. BMC Infect Dis 12: 111. 22574737 10.1186/1471-2334-12-111 PMC3447723

[39] Schauber J, Dorschner RA, Yamasaki K, Brouha B, Gallo RL (2006) Control of the innate epithelial antimicrobial response is cell-type specific and dependent on relevant microenvironmental stimuli. Immunology 118: 509–519. 16895558 10.1111/j.1365-2567.2006.02399.x PMC1782325

[40] Hamer HM, Jonkers D, Venema K, Vanhoutvin S, Troost FJ, et al (2008) Review article: the role of butyrate on colonic function. Aliment Pharmacol Ther 27: 104–119. 17973645 10.1111/j.1365-2036.2007.03562.x

[41] Guilloteau P, Martin L, Eeckhaut V, Ducatelle R, Zabielski R, et al (2010) From the gut to the peripheral tissues: the multiple effects of butyrate. Nutr Res Rev 23: 366–384. 20937167 10.1017/S0954422410000247

[42] Schauber J, Iffland K, Frisch S, Kudlich T, Schmausser B, et al (2004) Histone-deacetylase inhibitors induce the cathelicidin LL-37 in gastrointestinal cells. Mol Immunol 41: 847–854. 15261456 10.1016/j.molimm.2004.05.005

[43] Schwab M, Reynders V, Loitsch S, Steinhilber D, Schroder O, et al (2008) The dietary histone deacetylase inhibitor sulforaphane induces human beta-defensin-2 in intestinal epithelial cells. Immunology 125: 241–251. 18373608 10.1111/j.1365-2567.2008.02834.x PMC2561129

[44] Schwab M, Reynders V, Shastri Y, Loitsch S, Stein J, et al (2007) Role of nuclear hormone receptors in butyrate-mediated up-regulation of the antimicrobial peptide cathelicidin in epithelial colorectal cells. Mol Immunol 44: 2107–2114. 17055059 10.1016/j.molimm.2006.09.016

[45] Schauber J, Oda Y, Buchau AS, Yun QC, Steinmeyer A, et al (2008) Histone acetylation in keratinocytes enables control of the expression of cathelicidin and CD14 by 1,25-dihydroxyvitamin D3. J Invest Dermatol 128: 816–824. 17943182 10.1038/sj.jid.5701102

[46] Nakatsuji T, Kao MC, Zhang L, Zouboulis CC, Gallo RL, et al (2010) Sebum free fatty acids enhance the innate immune defense of human sebocytes by upregulating beta-defensin-2 expression. J Invest Dermatol 130: 985–994. 20032992 10.1038/jid.2009.384 PMC3057125

[47] Lalles JP, Bosi P, Smidt H, Stokes CR (2007) Nutritional management of gut health in pigs around weaning. Proc Nutr Soc 66: 260–268. 17466106 10.1017/S0029665107005484

[48] Iannitti T, Palmieri B (2011) Clinical and experimental applications of sodium phenylbutyrate. Drugs R D 11: 227–249. 21902286 10.2165/11591280-000000000-00000 PMC3586072

[49] Heidor R, Ortega JF, de Conti A, Ong TP, Moreno FS (2012) Anticarcinogenic actions of tributyrin, a butyric acid prodrug. Curr Drug Targets 13: 1720–1729. 23140283 10.2174/138945012804545443

[50] McGinty D, Letizia CS, Api AM (2012) Fragrance material review on benzyl butyrate. Food Chem Toxicol 50 Suppl 2S407–411. 22406567 10.1016/j.fct.2012.02.063

